# Skin wounds in a rural setting of Côte d’Ivoire: Population-based assessment of the burden and clinical epidemiology

**DOI:** 10.1371/journal.pntd.0010608

**Published:** 2022-10-13

**Authors:** Simone Toppino, Raymond T. A. S. N’Krumah, Bognan Valentin Kone, Didier Yao Koffi, Ismaël Dognimin Coulibaly, Frank Tobian, Gerd Pluschke, Marija Stojkovic, Bassirou Bonfoh, Thomas Junghanss

**Affiliations:** 1 Division Infectious Diseases and Tropical Medicine, Heidelberg University Hospital, Heidelberg, Germany; 2 Centre Suisse de Recherches Scientifiques en Côte d’Ivoire, Abidjan, Côte d’Ivoire; 3 Université Peleforo Gon Coulibaly de Korhogo, Korhogo, Côte d’Ivoire; 4 Université Félix Houphouët-Boigny d’Abidjan, Abidjan, Côte d’Ivoire; 5 Programme National de Lutte contre l’Ulcère de Buruli, Abidjan, Côte d’Ivoire; 6 Molecular Immunology Unit, Swiss Tropical and Public Health Institute, Basel, Switzerland; 7 University of Basel, Basel, Switzerland; The University of Hong Kong, CHINA

## Abstract

**Background:**

Data on the burden and clinical epidemiology of skin wounds in rural sub-Saharan Africa is scant. The scale of the problem including preventable progression to chronic wounds, disability and systemic complications is largely unaddressed.

**Methods:**

We conducted a cross-sectional study combining active (household-based survey) and passive case finding (health services-based survey) to determine the burden and clinical epidemiology of wounds within the Taabo Health and Demographic Surveillance System (HDSS) in rural Côte d’Ivoire. Patients identified with wounds received free care and were invited to participate in the wound management study simultaneously carried out in the survey area. The data were analysed for wound prevalence, stratified by wound and patient characteristics.

**Results:**

3842 HDSS-registered persons were surveyed. Overall wound prevalence derived from combined active and passive case finding was 13.0%. 74.1% (403/544) of patients were below the age of 15 years. Most frequent aetiologies were mechanical trauma (85.3%), furuncles (5.1%), burns (2.9%) and Buruli ulcer (2.2%). Most wounds were acute and smaller than 5 cm^2^ in size. 22.0% (176/799) of wounds showed evidence of secondary bacterial infection. 35.5% (22/62) of chronic wounds had persisted entirely neglected for years. Buruli ulcer prevalence was 2.3 per 1000 individuals and considerably higher than expected from an annual incidence of 0.01 per 1000 individuals as reported by WHO for Côte d’Ivoire at the time of the study.

**Conclusions:**

Skin wounds are highly prevalent in rural West Africa, where they represent a widely neglected problem. The HDSS-based survey with combined active and passive case finding adopted in this study provides a better estimate than school- and health institution-based surveys which underestimate the frequency of skin wounds and, particularly, of neglected tropical diseases of the skin, such as Buruli ulcer and yaws. A comparison with country-specific WHO data suggests underreporting of Buruli ulcer cases.

**Trial registration:**

Registration at ClinicalTrials.gov NCT03957447.

## Author summary

Data on the burden and clinical epidemiology of skin wounds in rural sub-Saharan Africa is scant. The scale of the problem including preventable progression to chronic wounds, disability and systemic complications is largely unaddressed.

We conducted a cross-sectional study combining active (household-based survey) and passive case finding (health services-based survey) to determine the burden and clinical epidemiology of wounds within the Taabo Health and Demographic Surveillance System (HDSS) in rural Côte d’Ivoire. Patients identified were invited to participate in the wound management study simultaneously carried out in the survey area.

We surveyed approximately 4000 HDSS-registered persons and found a high overall wound prevalence (13.0%), predominately in children. Mechanical trauma was the leading cause, followed by furuncles, burns and Buruli ulcer. Most wounds were acute and had a size of less than 5 cm^2^, but a substantial proportion was large, complicated, or chronic, some of the latter persisting neglected for years.

The HDSS-based survey with combined active and passive case finding adopted in this study provides a better estimate than school- and health institution-based surveys which underestimate the frequency of skin wounds and, particularly, of neglected tropical diseases of the skin, such as Buruli ulcer and yaws. A comparison with country-specific WHO data suggests underreporting of Buruli ulcer cases.

## Introduction

Wounds have a very high economic and social impact in high-income societies, where the clinical epidemiology is well described [1]. Despite growing efforts to study and define the global burden of skin diseases, published data on skin wounds in sub-Saharan Africa (SSA) are scarce [2]. The Global Burden of Disease Study 2019 provides the most recent comprehensive analysis of morbidity and mortality in SSA [3]. Wounds are not explicitly addressed, but data is provided on common causes of wounds, such as injuries. In 2019, injuries caused 6.8% and 6.1% of all Disability-Adjusted Life Years (DALYs) in SSA and Côte d’Ivoire, respectively. The leading causes of injuries were road accidents, followed by interpersonal violence, self-harm, falls and burns [4]. A multi-country study in SSA reported a serious injury in the previous 12 months in 17.7% of the surveyed population, with road accidents and sharp instruments as main causes [5]. Injuries affect population subgroups disproportionately. More than 30% of all injury-related DALYs registered in Côte d’Ivoire in 2019 occurred among children younger than 14 years of age [3]. A review on burns found that 83.6% of all reported burns in SSA occurred among children below the age of 10 years [6].

Another common cause of wounds in SSA are neglected tropical diseases of the skin (skin NTDs) that commonly present with skin ulcerations, such as BU, yaws, leprosy, scabies and late stage lymphatic filariasis [7,8], also endemic in Côte d’Ivoire. In SSA current estimates of skin ulcer prevalence rely on data of national skin NTD control programs. Retrospective BU data reported for the Tiassalé district of Côte d’Ivoire revealed an average annual incidence rate of 39 BU cases per 100,000 people across the entire district surveyed and 234 BU cases per 100,000 people in the communities near the new hydroelectric Bandama River dam, pointing at clustering of BU [9]. According to country-specific data from WHO, 251 new BU cases were reported in Côte d’Ivoire through surveillance data from national healthcare systems in 2019 [10]. In a study carried out in Cameroon up to 73% of patients with skin ulcers presenting to a BU reference centre finally received an alternative diagnosis [11]. Mass outreach programs report an abundance of neglected wounds other than those specifically addressed by the program [12,13]. Two school-based cross-sectional surveys on skin diseases in general, in which wounds were not specifically addressed, reported a wound prevalence of 2% or less [14,15]. A study on yaws in Cameroon investigated the selection bias of various survey methods [12].

We present here the results of a study combining a household- and a health services-based survey to assess the burden and clinical epidemiology of skin wounds in a rural community of Côte d’Ivoire within a Health and Demographic Surveillance System (HDSS).

## Methods

### Ethics statement

The study protocol has been approved by the Comité National d’Éthique de la Recherche (CNER) of Côte d’Ivoire (N° 081-18/MSHP/CNESVS-km) and the Ethical Review Board of Heidelberg University Hospital (N° S-797/2018). The study has been registered at ClinicalTrials.gov under the registration number NCT03957447. Written consent was obtained from all participants aged 18 years and older, and from parents, caretakers, or legal representatives of participants younger than 18 years.

### Study setting

The study took place in the Ahondo Health Area, Tiassalé district, Côte d’Ivoire which is part of Taabo HDSS [9,16,17]. Geographically, the Ahondo Health Area is in the south of Côte d’Ivoire in the sub-prefecture of Taabo, in a transition zone between savannah and forest and close to an artificial lake. It has a population of approximately 4,200 people, who mostly depend on subsistence agriculture. The Ahondo Health Centre was the only health centre active in the area at the start of the study and is located 15 km away from the closest district hospital in Taabo. In December 2019 a second health centre in the nearby village of Sahoua became active but did not cover wound management during the study period. BU is highly prevalent in the Ahondo village area with an average annual incidence of 370 cases per 100,000 inhabitants reported for the 2005–2010 period [9].

### Study design and participants

We conducted a cross-sectional study combining active (household-based survey) and passive case finding (health services-based survey) to determine the prevalence and clinical epidemiology of wounds in a rural community.

A wound management study simultaneously implemented in the surveyed area offered high quality care for the patients identified at the survey, contributed detailed descriptions and the final diagnoses of the wounds [18].

**Fig 1:**
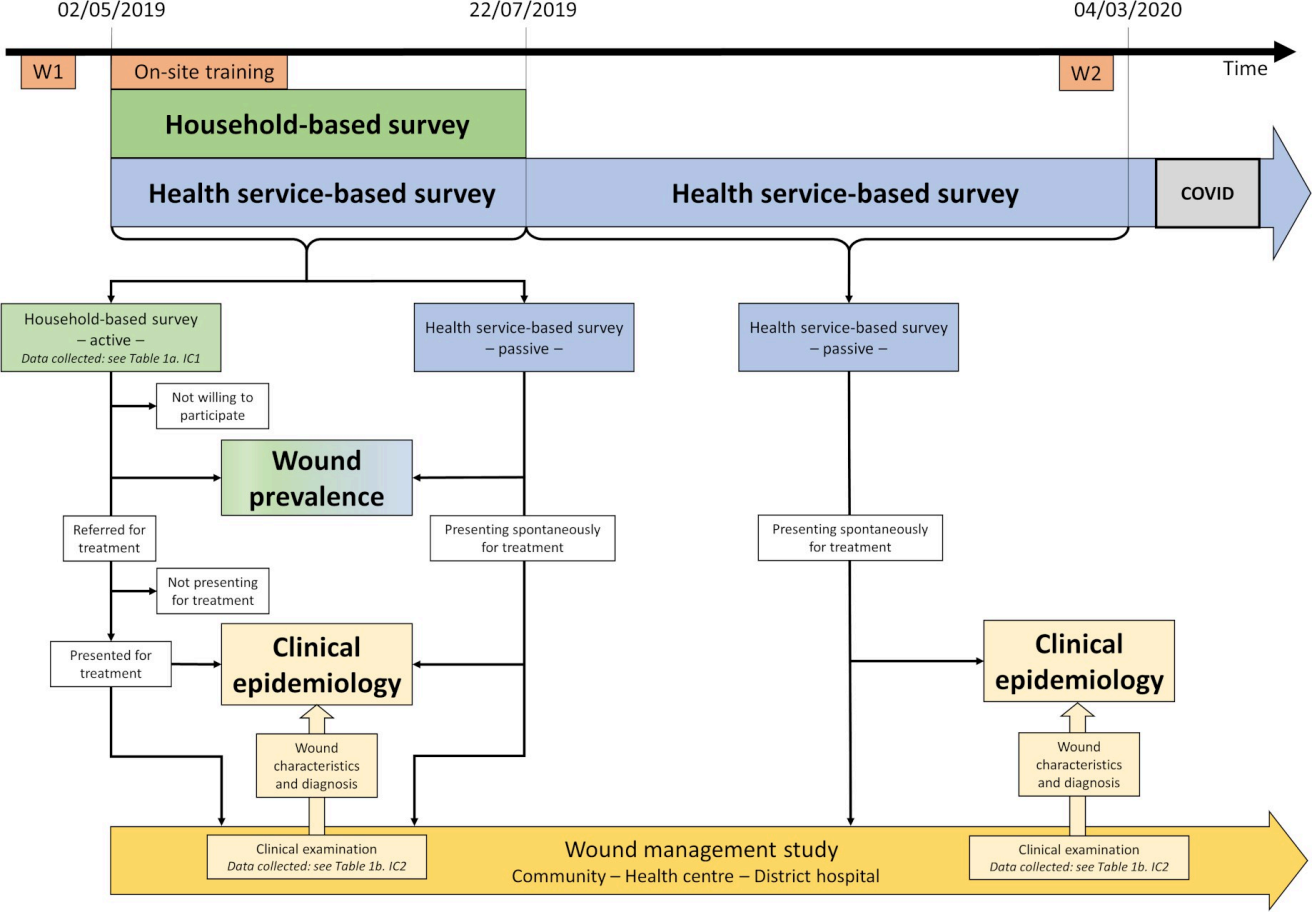
Study flow chart: interaction between household- and health services-based surveys and wound management study. Patients with wounds were actively (household-based survey) and passively (health services-based survey) identified in Ahondo Health Area which is part of the Taabo Health and Demographic Surveillance System (HDSS) to determine the wound prevalence and the clinical epidemiology of wounds in a rural setting of West Africa. This was combined with an observational wound management study at three health care levels–community, health centre and district hospital–aiming at early identification and treatment of wounds [18]. This approach secured appropriate treatment for all patients identified during the wound surveys. W1 and W2: Training workshops of nurses, assistant nurses, and community health workers (CHW). IC1 and IC2: Informed consent for cross-sectional study and wound management study respectively.

The health services-based survey started on May 2^nd^,2019 and has continued since then, the household-based survey ran between May 2^nd^ and July 22^nd^, 2019. The dataset was finalized on March 4^th^, 2021, for long-term observation of complicated chronic wounds. For details see Fig 1.

Wound was defined as broken skin barrier and all individuals with wounds were eligible for the surveys. There were no exclusion criteria.

In the household-based survey (active case finding) HDSS enumerators familiar with the local community enrolled patients with wounds, collected a set of baseline data (see Table 1) and encouraged the patients identified with wounds to attend the health services for treatment. They informed patients about the ongoing wound management study. The speed at which the household-based survey moved forward was tuned to the health services capacity to cope with the number of patients identified.

**Table 1:** Data collected during the household-based survey and the health services-based survey / wound management study.

A. Household-based survey data	B. Health services-based survey data / wound management study
• HDSS ID • Age • Sex Wound assessment • Wound photograph • Time period since first noticed • Associated pain • Anatomical site • Presumed cause • Past treatment	• HDSS ID • Age • Sex • Weight* • Height* • Body temperature • Blood pressure* • Heart rate* • Presence of: ○ Generalized erythema* ○ Pallor* ○ Jaundice* ○ Oedema (not associated with the wound) * ○ Enlarged and tender peripheral lymph nodes* Wound assessment • Wound photograph • Time period since first noticed • Associated pain • Anatomical site • Size (maximum length and width) * • Edge (undermined, collapsed or raised) * • Signs and symptoms of infectious complications • Past treatment

Items marked with * were not included in the simplified forms used for wounds treated in the community. Signs and symptoms of infectious complications are listed in the section ‘Wound infection and complications’.

Local healthcare personnel (nurses, assistant nurses, community health care workers (CHWs)) of the wound management study took the medical history, performed the clinical examination (see Table 1) and made a presumptive diagnosis (see section ‘Wound diagnosis’) of patients spontaneously presenting with wounds to Ahondo Health Centre, the wound management unit of Taabo district hospital (WMU) or to CHWs in the community (health services-based survey / passive case finding) and of the patients referred from the household-based survey (active case finding).

The presumptive diagnosis was the starting point of the wound management study and after confirmation the endpoint of the clinical epidemiological / wound burden study [18].

Data collected on paper CRFs are shown in Table 1. A simplified CRF version was used in the community by CHWs (simple wounds).

Criteria on which wound diagnosis was based are described in the section ‘Wound diagnosis’.

### Wound diagnosis

**Fig 2:**
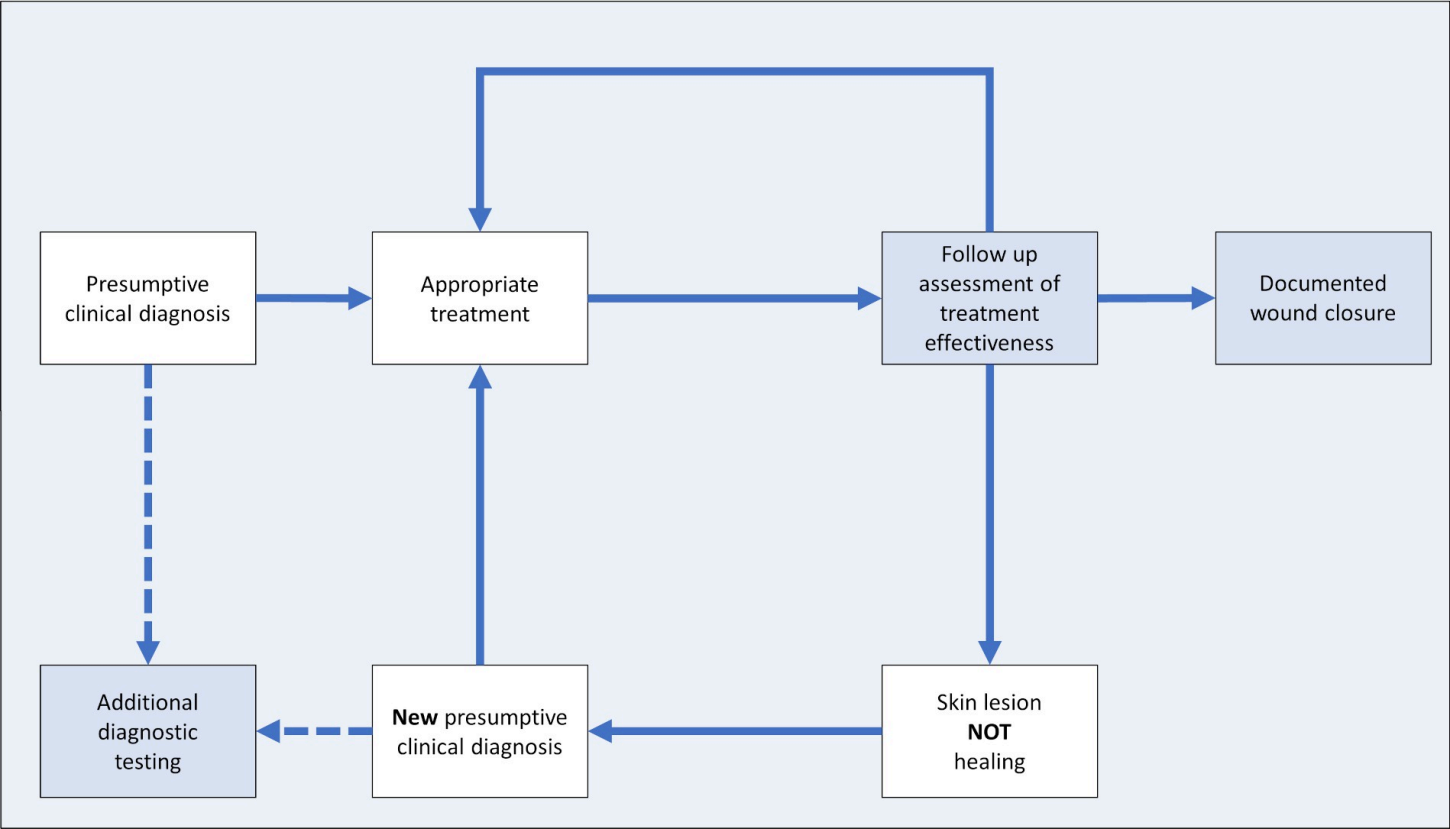
Diagnostic cycle to review presumptive diagnosis and treatment. Assessment steps leading to either confirmation of a presumptive clinical diagnosis or revision (light blue-coloured boxes). Clinical investigators reviewed selected cases on-site or via mobile phone consultations and performed a systematic review of each case based on clinical data and wound photographs. In case of treatment failure, the cause of treatment failure was analysed and it was decided if the current diagnosis could be maintained, but treatment had to be adapted / reinforced / intensified or the diagnosis and treatment had to be revised. If it deemed indicated additional laboratory investigations and imaging (underlying bone disease—osteomyelitis) were ordered; this was done routinely for the specific presumptive diagnoses BU, tuberculosis, yaws, and osteomyelitis conducted at local facilities, national and international laboratories as appropriate.

The presumptive diagnosis and treatment installed by the local healthcare personnel (nurses, assistant nurses, CHWs) of the wound management study were reviewed by the clinical investigators. If treatment failure was suspected at follow-up visits the diagnosis was again reviewed and if necessary revised (see diagnostic cycle—Fig 2). Except for aetiologies requiring disease-specific treatment, such as BU or yaws, a diagnosis was considered confirmed when a wound healed under the treatment initiated according to the initial presumptive diagnosis, assuming aetiologies requiring disease-specific treatment would not have healed under basic wound management alone.

**Fig 3:**
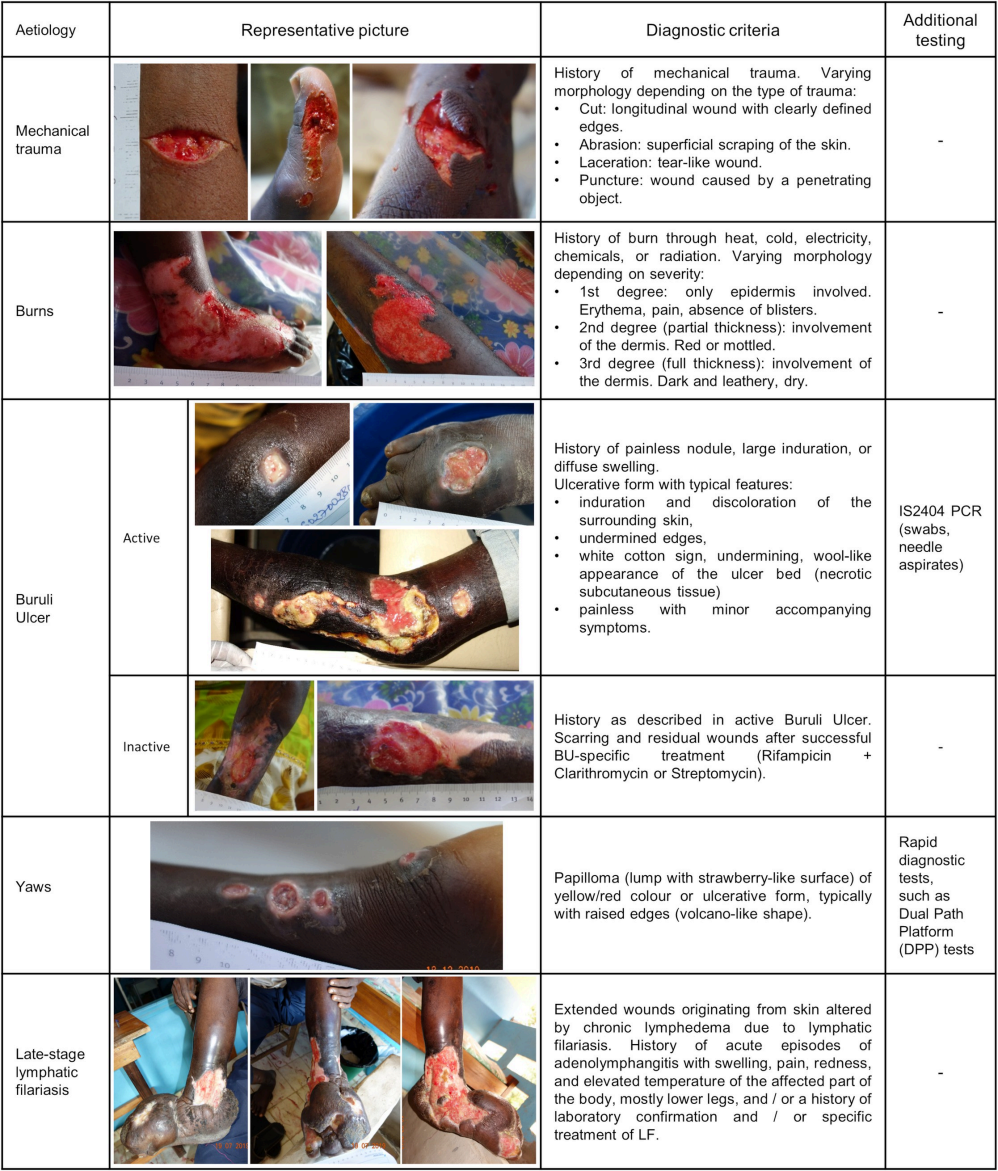
Diagnostic criteria and representative images of the main wound aetiologies affecting patients in the study population. Diagnostic criteria are based on the WHO Manual ‘Recognizing Neglected Tropical Diseases through Changes on the Skin’ (8), and a manual assembled by the project team and provided to the local healthcare personnel at the training workshops. All representative photographs were taken from study patients.

Diagnostic criteria and representative images of the main aetiologies in the study population are shown in Fig 3. Wound closure was defined as closure of the wound either by epithelization or a stable scar. The wound development was regularly documented by photography; wound closure by photography and / or direct assessment by healthcare personnel or, exceptionally, by indirect assessment by healthcare personnel, i.e. statement of the patient or legal representative for children below 18 years of age or of a close relative (parents or siblings). Wounds were considered acute if reported time since first noticed was less than 4 weeks, chronic if more than 4 weeks.

### Wound infection and complications

Wound assessment was based on the criteria adapted from [19]. Local and deep infection were defined by the presence of three or more criteria respectively (see Table 2). Systemic infection was defined by presence of deep infection and fever (body temperature ≥ 38.5°C), e.g. abscess, erysipelas, cellulitis and non-hematogenous osteomyelitis.

**Table 2:** Clinical criteria for local and deep secondary bacterial infection.

Local infection	Deep infection
• Non-healing • Increase in exudate or purulent exudate • Red friable granulation tissue • Debris • Foul odour	• Increase in wound size • Elevated temperature of the perilesional skin • Bone involvement • New areas of skin breakdown • Increase in exudate or purulent exudate • Erythema or oedema of perilesional skin • Foul odour

Enlarged and tender peripheral lymph nodes were considered as additional sign of deep and systemic infection.

### Data management and analysis

Following quality check by the clinical investigators data were double-entered into a RedCap database [20,21] and cleaned. The data were analysed for wound prevalence, stratified by wound characteristics (size, aetiology, presumptive and confirmed diagnosis, time since appearance, site, evidence for secondary bacterial infection, and associated pain), patient characteristics (listed in Table 1B) and health service level where wounds were primarily treated (community, Ahondo Health Centre, WMU of Taabo district hospital).

Wound prevalence analysis was calculated based on the wound events leading to the detected wounds. Wound event was defined as an injury (mechanical trauma, burn, etc) or a specific pathology (BU, yaws, etc) leading to one or multiple wounds. Wounds enrolled on the same date and attributed to the same aetiology were considered as one wound event. Specific aetiologies, such as BU or yaws, that could lead to multiple wounds over time were considered a single wound event. The rationale for this classification is based on the fact that a study participant can be enrolled more than one time into the study, e.g. initially with a BU and sometime later with a traumatic wound due to a road accident. Statistical analysis was performed with RStudio (R Core team, Version 1.4.1106). Assessment of nutritional status was based on WHO recommendations [22,23].

## Results

### Patient and population characteristics

A total of 561 patients with wounds were enrolled in the study, 410 during the period of combined active and passive case finding (May 2^nd^—July 22^nd^, 2019) and 151 during the period of passive case finding only (July 23^rd^, 2019 –March 4^th^, 2020).

The household-based survey covered 100% of the Ahondo Health Area population. At the time of the last HDSS survey (August—December 2018) 4245 people were registered in the Ahondo Health Area. Among these, 3803 persons could be surveyed, 417 had moved out of the village and 25 were deceased. 39 new individuals with wounds could be registered during the survey, leading to a total study population of 3842. 767 households were surveyed, with an average household size of 5.0 people.

**Fig 4:**
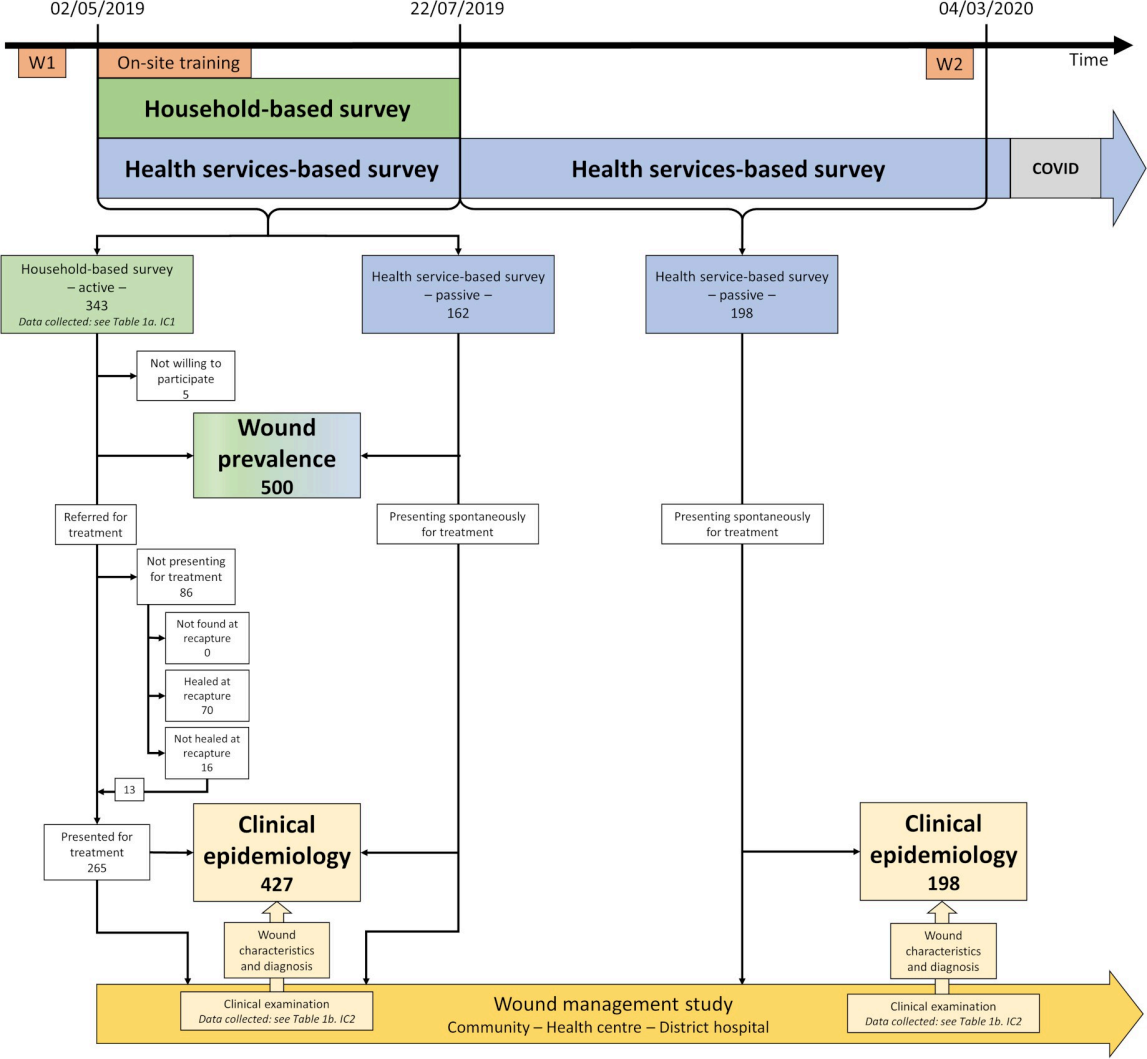
Enrolment of wound events during household- (active case finding) and health services-based survey (passive case finding). Wound event was defined as an injury (mechanical trauma, burn, etc) or a specific pathology (BU, yaws, etc) leading to one or multiple wounds. Wounds enrolled on the same date and attributed to the same aetiology were considered due to the same wound event. Specific aetiologies, such as BU or yaws, that could lead to multiple wounds over time were considered as a single wound event.

Fig 4 shows the distribution of wound events enrolled during the study period.

**Fig 5:**
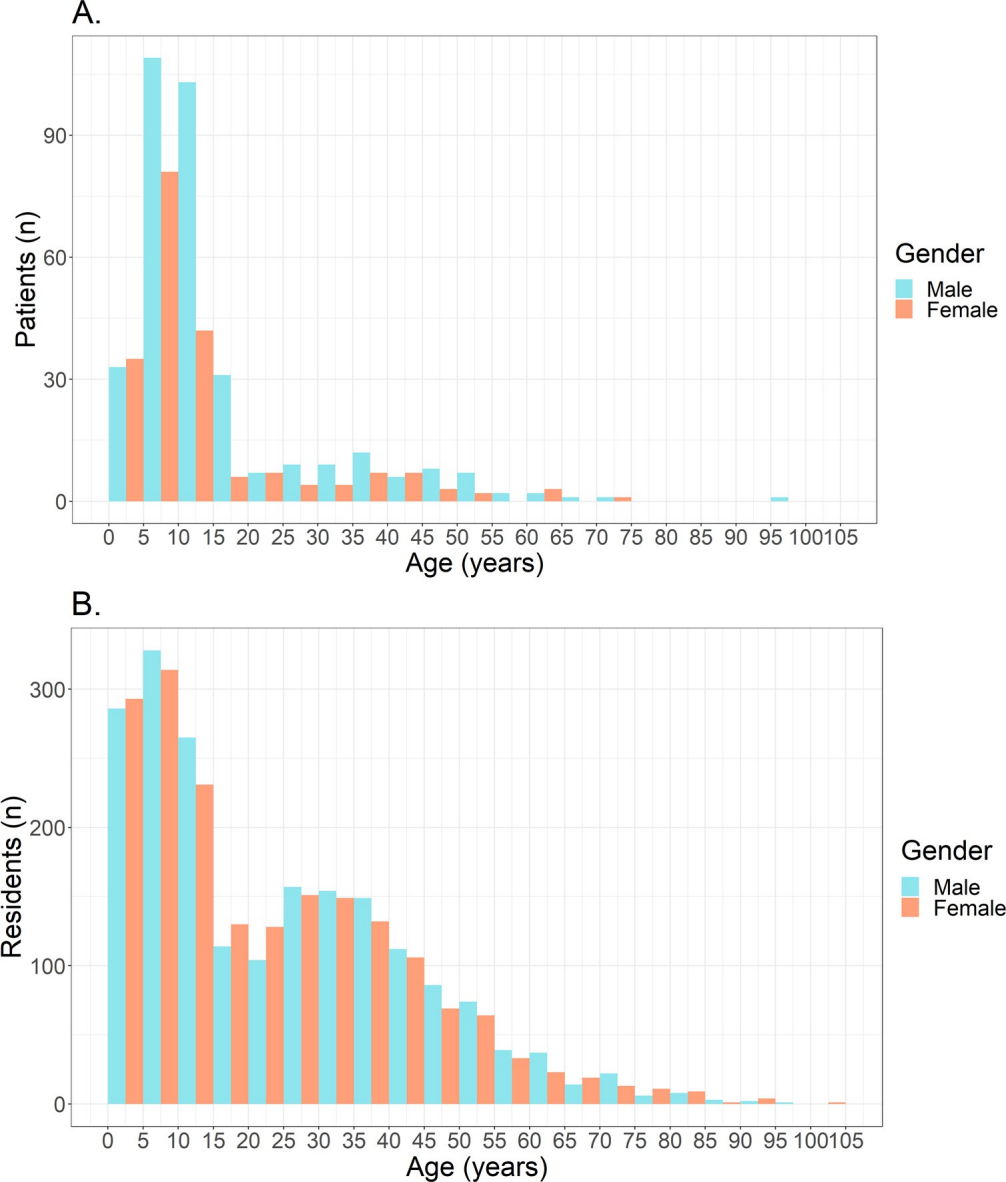
Age distribution by gender of the patient population (A) and of the Ahondo Health Area population (B).

Median age of the patient population was 10.0 years (mean 14.6, IQR 7.0–15.0). 74.1% (403/544) of patients were below the age of 15, with the age group 5–10 being the most represented. 63.0% of patients were male, corresponding to a male to female ratio of 1.7. In the Ahondo Health Area 44.7% (1717/3842) of the population is younger than 15 years, with a median age of 19 years (mean 23.2, IQR 7.0–36.0). The male to female ratio in the population of the Ahondo Health Area is 1.04. Fig 5A and 5B show the age distribution of the patient population and of the Ahondo Health Area population.

Among 340 patients presenting for treatment at the Ahondo Health Centre or at the WMU of Taabo District hospital, weight and height were collected for 214 patients. Among adults 4.8% (2/42) were underweight, 11.9% (5/42) overweight and 4.8% (2/42) obese. Among children between 5 and 19 years of age 6.1% (9/148) and 6.8% (10/148) suffered from thinness and severe thinness respectively; 2.0% (3/148) were obese. Among 18 children younger than 5 years, the proportions of stunting, underweight and wasting were 22.2%, 11.1% and 11.1%, respectively.

### Wound characteristics

Wound prevalence in the Ahondo Health Area was 13.0% (95% confidence interval 12.0–14.1) based on the data collected during combined active and passive case finding. Most of the 561 patients (81.3%) had one wound event, 13.9% had two, 4.8% had three or more. A total of 923 wounds were included in the wound management study and 903 (903/923, 97.8%) were considered for analysis of the clinical epidemiology, 20 wounds were excluded because of missing data on aetiology. Overall, 49 of 923 wounds (5.3%) were lost to follow-up, among these, 2 wounds belonged to a patient deceased during the study period. 4 wounds had not closed and were still under treatment at end of the study period (March 4^th^, 2021).

Table 3 shows the distribution of wound aetiologies according to presumptive clinical diagnoses at enrolment and the number of confirmed diagnoses at each level of assessment. Presumptive diagnosis was confirmed by documented wound closure in 94.2% (851/903) of cases. Presumptive clinical diagnosis at enrolment was revised in 4 (0.5%) cases following assessment by the wound management team (Table 3).

**Table 3:** Distribution of wound aetiology, presumptive clinical diagnosis at enrolment and confirmation.

	Presumptive diagnosis at enrolment	Confirmation / change of presumptive diagnosis at follow-up review			Documented wound closure
Aetiology (Wounds)	N	N			N (%)
Mechanical trauma	768	766			733 (95.7)
		- 1 → - 1 →	BU Diphtheria		
Furuncle*	42	41			35 (85.4)
		- 1 →	BU		
Burn	32	32			30 (93.8)
BU	22	25			23 (92.0)
			Mechanical trauma Chronic wound of unknown origin Furuncle	→ +1 → +1 → +1	
Yaws	14	14			14 (100)
Chronic wound of unknown origin	9	8			2 (25.0)
		- 1 →	BU		
Inactive BU	5	5			2 (40.0)
Surgical	3	3			3 (100)
Lymphatic filariasis	3	3			3 (100)
Cellulitis / Erysipelas	3	3			3 (100)
Diphtheria	0	1			1 (100)
			Mechanical trauma	→ +1	
Abscess	1	1			1 (100)
Animal bite	1	1			1 (100)
Total	903	903			851 (94.2)

Diagnosis revisions are shown both in the row of the initial diagnosis (subtracted and moved to revised diagnosis) and the row of the wound category as revised (the revised wounds are added with reference to the original diagnosis). The proportion of wounds with documented wound closure refers to the total of the confirmed diagnoses. Laboratory testing for BU (IS2404 PCR) was performed on 11 patients (with 20 wounds) classified as BU: 9 patients (with 18 wounds) tested positive; 2 patients (with 2 wounds) tested negative. For 5 patients (with 5 wounds) with suspected BU no laboratory testing was available, 3 of which were clinically unequivocal. Cases of inactive BU were not tested. Additional testing for yaws (DPP) was performed on 1 of 4 patients, who tested positive. * Cases of deep folliculitis were included in the diagnostic category “Furuncle”.

Table 4 shows the aetiology distribution based on wound events and the corresponding prevalence calculated on the combined household- and health services-based survey. Only confirmed diagnoses were considered.

**Table 4:** Estimated wound prevalence and aetiology distribution stratified by study period.

	Combined household- and health services-based survey			Health services-based survey		Whole study period	
Aetiology (Wounds events)	N	%	Prevalence per 1000 inhabitants (95% CI)	N	%	N	%
*Mechanical trauma*	354	85.3%	92.1 (83.0-101.3)	168	86.6%	522	85.7%
Furuncle*	21	5.1%	5.5 (3.1-7.8)	2	1.0%	23	3.8%
*Burn*	12	2.9%	3.1 (1.4-4.9)	12	6.2%	24	3.9%
*Buruli Ulcer*	9	2.2%	2.3 (0.8-3.9)	7	3.6%	16	2.6%
*Yaws*	4	1.0%	1.0 (0.02-2.1)	0	-	4	0.7%
*Chronic wound of unknown origin*	6	1.4%	1.6 (0.3-2.8)	2	1.0%	8	1.3%
*Inactive BU*	4	1.0%	1.0 (0.02-2.1)	0	-	4	0.7%
*Lymphatic filariasis*	1	0.2%	-	0	-	1	0.2%
*Surgical*	0	0.0%	-	3	1.5%	3	0.5%
*Cellulitis / Erysipelas*	1	0.2%	-	0	-	1	0.2%
*Diphtheria*	1	0.2%	-	0	-	1	0.2%
*Abscess*	1	0.2%	-	0	-	1	0.2%
*Animal bite*	1	0.2%	-	0	-	1	0.2%
*Subtotal (confirmed diagnoses)*	415	100%	-	194	100%	609	100%
Missing data**	85	-	-	4	-	89	-
*Total*	500	-	130.1 (119.5-140.8)	198	-	698	-

Aetiology distribution and wound prevalence were based on wound events. Estimated wound prevalence was calculated for the combined household-based survey (active case finding) and health services-based survey (passive case finding) and only for aetiologies with 4 or more wound events. * Cases of deep folliculitis were included in the diagnostic category “Furuncle”. ** Diagnoses could not be confirmed either because patients with wounds detected during the house-to-house survey did not present to the health services to receive free treatment despite encouragement (n = 73) or because data on follow-up were missing (n = 16).

Wound size was documented for 532 / 571 of the wounds that had full assessment at the Health Centre / WMU of Taabo district hospital. 87.2% (464/532) were smaller than 5 cm^2^, 6.8% (36/532) had a size between 5 and 15 cm^2^, 6.0% (32/532) were larger than 15 cm^2^. 352 wounds (38.1%) were enrolled in the community with simplified CRFs (no size measurements; see Table 1) which were small, uncomplicated, and treatable by CHWs and were not included in this analysis.

Data on time since the wound was first noticed was available for 841 wounds: 70.7% were acute, 7.4% were chronic (i.e. reported time since first noticed was more than 4 weeks) and in 21.9% of cases the patient could not recall when the wound appeared. 22 chronic wounds were reported to have been lasting for several years. 22.0% (176/799) of wounds showed evidence of infection at enrolment. Local infection was most common (14.9% of all wounds) followed by deep (4.1%) and systemic infection (3.0%). At enrolment most patients (76.8%, 624/813) did not report current pain associated to the wound. 35.7% (10/28) of BU-related wounds were reported as painful at enrolment.

### Population subgroups

Wound event-based prevalence was highest in the age groups 5–10 and 10–15 years old, calculated as 29.4% (95% CI 25.9–33.0) and 27.4% (95% CI 23.5–31.3), respectively. Males had overall higher wound prevalence than females (15.8% vs 9.7%). This difference was highest in the age groups 10–15 and 15–20 years old. Traumatic wounds were most prevalent in the age groups 5–10 and 10–15 years old (21.0% [17.9–24.2] and 21.4% [17.8–25.0] respectively) and more prevalent in males than females (11.8% vs 6.4%), particularly in age groups 5–10, 10–15 and 15–20 years old. 11 of 16 patients with active BU were younger than 15 years old, 12 of 16 were males.

## Discussion

Wound aetiologies in rural West Africa include injuries, such as mechanical traumas and burns, and infectious aetiologies, such as skin NTDs. The clinical epidemiology of wounds varies across regions depending on geographical, environmental, and socio-economic factors. National control programs for skin NTDs and independent studies provide data on the burden of these diseases in endemic countries [24–28]. Wounds of other aetiologies are often neglected, particularly in rural areas, and comprehensive data on the burden of skin wounds in rural sub-Saharan Africa are scarce [11,12,24,25,29,30].

Most of the available data originate from school- or health services-based surveys [10,12,14,25,29]. National control programs of skin NTDs rely mostly on passive surveillance data from national healthcare systems. Without awareness campaigns that involve the communities, this strategy has limitations and is associated with underreporting [12,26]. School-based surveys focus exclusively on children and are influenced by schooling rates and–particularly for stigmatizing diseases such as skin NTDs–by social isolation of affected patients [12,13,31]. To overcome these selection biases, we conducted simultaneously a health services-based survey for passive case finding and a household-based survey for active case finding in a population under HDSS surveillance. We adopted a horizontal approach and included all wounds, defined as broken skin barrier, independently of their aetiology. This is in line with the WHO-recommended integrated strategy for skin NTDs [25] and extends the concept to non-NTD aetiologies.

To ensure appropriate treatment of all detected wounds, we combined our surveys with a community-based wound management study at three health care levels (community, health centre, district hospital) and offered free treatment to all identified patients. Detected wounds received a presumptive diagnosis by local healthcare personnel trained in wound management according to national recommendations and WHO standards [8,32] and all patients were followed up longitudinally. Based on careful reassessment of treatment effectiveness and on review by clinical investigators, initial diagnoses were either confirmed or revised during follow-up. Training on wound assessment and on identification of specific aetiologies and complications, such as secondary bacterial infections, led to high therapeutic success rate. The treatment selected on the basis of the presumptive diagnoses provided by local healthcare personnel resulted in 99.5% of cases in wound closure [18]. Specific aetiologies (BU and yaws) received additional diagnostic tests in national and/or international laboratories. With our combined approach of a household- and health services-based survey we could overcome the biases of purely institutional or population-based cross-sectional surveys. Additionally, in population-based cross-sectional surveys where teams move quickly from house to house it is difficult to arrive at validated diagnoses and to ensure that appropriate care is provided to all identified patients. This was secured with the wound management study, which went along with the surveys.

We surveyed 3842 HDSS-registered people, covering a high proportion of the approximately 4,200 people living in the study area. Taabo HDSS has been collecting data in the study area since 2009 and benefits from high acceptance by the local population. This allowed us to achieve a high coverage percentage of the resident population and to perform a denominator-based epidemiological analysis. Wound prevalence was calculated based on wound events. A wound event was defined as an injury (mechanical trauma, burn, etc) or a specific pathology (BU, yaws, etc) leading to one or multiple wounds. The period wound prevalence derived from combined active and passive case finding was 13.0%. Active case finding was tuned to the healthcare services capacity to cope with the number of patients identified. As a result, combined active and passive case finding lasted 81 days in total. Wound prevalence was highest (28.6%) among school-age children (between 5 and 15 years old). For comparison, two school-based cross-sectional surveys on skin diseases–which did not specifically address skin wounds—found a wound prevalence of 2% or less [12,13]. Relying on school-based surveys for assessment of the wound burden could therefore lead to underestimation [12].

Most wounds were acute (70.7%) and small (87.2% with a surface area less than 5 cm^2^) and the most common aetiology was mechanical trauma (85.3%). These wounds are mostly due to minor injuries, such as falls, that are frequently encountered among children, and contributed substantially to the higher wound prevalence in this population group. If treated early, these wounds heal quickly under basic standard wound management, as shown in the results of our wound management study [18]. Without prompt treatment, complications can develop, particularly in settings where access to water, sanitation and hygiene services is limited. Among wounds detected in the study, 22.0% showed signs of secondary bacterial infection, most commonly local infection (14.9%), followed by deep (4.1%) and potentially life-threatening systemic infections (3.0%). Lack of treatment and recurring secondary bacterial infections lead to arrested wound healing and wound chronification. 43.5% of chronic wounds were due to mechanical trauma and could have potentially been prevented by early detection and treatment.

Furuncles were the second most frequent aetiology, causing 5.1% of wound events detected during the combined household- and health services-based survey, with a calculated prevalence of 5.5 per 1000 individuals. A school-based survey for skin diseases in Côte d’Ivoire has found folliculitis representing 1.8% of detected pathologies, with a calculated prevalence of less than 1% [14]. A multi-country study among schoolchildren reported for Ghana prevalence estimates for superficial bacterial infections including folliculitis, furuncles, and impetigo between 4.3% and 5.8% [15]. These infections were also detected more frequently in rural areas and among children with lower socio-economic level, such as in the setting of our study [15].

Burns caused 2.9% of wound events detected during the combined household- and health services-based survey, with a calculated prevalence of 3.1 per 1000 individuals. Burns represent an important cause of mortality and morbidity in sub-Saharan Africa, with children being most affected and bearing the highest burden of burn deaths worldwide [6,33]. Data on burns mostly rely on health services-based surveys and prevalence estimates at the community level are lacking. Most burns detected in our study were not severe and could be treated at the community or health centre level. Minor burns are likely to be underreported in studies based on passive surveillance data from national healthcare systems, but, similarly to furuncles, have the potential for developing complications and chronification if left untreated. In West Africa scalds and flames are the most common causes of burns [6,34], which could be addressed by targeted prevention strategies tailored to the context of rural communities [35].

BU was the fourth most common wound aetiology. The study area is known to be endemic for BU [9], but we detected more cases than expected from current national estimates for Côte d’Ivoire, where WHO has reported since 2009 steadily declining numbers of new cases per year. In 2019, 251 new BU cases were reported for this country of 25,717,000 people [10,36]. Within 10 months of observation, we could detect 16 active BU cases among the 3,842 residents of the Ahondo Health Area enrolled in our study and calculated a BU prevalence for the study area of 2.3/1000 (95% CI 0.8–3.9/1000). These results also differ significantly from a recent epidemiological study on the incidence of skin diseases in Côte d’Ivoire, where a school-based cross-sectional survey did not find any case of BU in another area of the country [14]. This substantial difference may be explained by a composite of geographical clustering [26] and selection bias, as children affected by BU may not attend school because of stigmatization and social isolation [12,31]. This consideration is particularly relevant for studies assessing the burden of BU, as this pathology mostly affects children [26,37]. Consistent with these findings, 68.8% of BU patients detected in our study were children below the age of 15 years. Most cases were classified as WHO category I, but we also identified three severe cases of WHO category III, two of which have persisted neglected for several years. Because of several factors, including lack of community awareness, initial misdiagnosis, social isolation, financial constraints and many affected patients first resorting to traditional healers, BU cases often reach medical attention late in the course of the disease [26]. Within our study, 43.8% (7/16) of BU wounds were reported as chronic. This finding is most likely due to active case finding, that allowed detection of many cases in the early stages of disease. Consistently with the literature, most BU wounds (64.3% 18/28) were painless at enrolment [38], although pain was reported in most wounds during treatment and wound dressings, similar to the findings of a previous BU study [24].

We did not encounter wounds caused by snakebites during this study period and only surprisingly few in the continuing project. To rule out that patients may not want to disclose this aetiology or may not be aware of circumstances suggestive of snake bites we now added more questions on snakebites in the continuing surveys.

This study has some limitations. Wound prevalence estimates did not consider seasonality, as surveys were conducted only during the rainy season. Fluctuations in incidence across seasons have been described for burns and BU [6,26,34] and may affect other aetiologies. Previous studies have also highlighted that case detection may be affected by migratory patterns related to agricultural work [12]. In our ongoing wound management project, we will continue with the household-based surveys alternating between dry and rainy seasons to investigate seasonal variations.

Similarly, entries (immigration and new births) and exits (emigration and deaths) of residents within the study area have been recorded between HDSS surveys. We surveyed all HDSS-registered individuals of the study area and removed emigrated or deceased individuals from the resident list of the last HDSS survey. We registered newly arrived individuals with wounds who fulfilled HDSS criteria for residency within the area but did not capture all newly arrived individuals without wounds as this would have demanded more resources as available in a standard HDSS where the newly arrived individuals are only captured twice a year. Therefore, the number of residents at the time of the wound survey may have been underestimated. Based on an average of 70.7 entries per month between HDSS surveys in the 2016–2018 period, our prevalence estimates could be overestimated, leading to an overall wound event prevalence of 11.4% instead of 13.0%.

Our household-based survey relied on patient self-reporting and was therefore influenced by the wound perception of the local population. During our surveys we did not specifically address obstetric wounds and wounds due to female genital mutilations (FGM). None have been reported during the household-based survey. Caesarean section is the most common operative procedure performed in sub-Saharan Africa and is associated with a high incidence of wound infection [39]. Although illegal, FGMs are still practiced in Côte d’Ivoire: according to UNICEF, in 2016 10% of girls aged 0 to 14 years had undergone FGMs, with higher prevalence reported in rural areas [40]. FGMs cause severe trauma and morbidity, and death due to complications, but girls and women who have undergone FGM often delay or do not seek help because of several factors, including shame and fear of legal persecution [41]. We plan to specifically address these wound aetiologies in the ongoing project, through the cooperation with the local midwife.

In Côte d’Ivoire, laboratory confirmation of suspected BU and yaws is taken care of by the national control programs. We made use of the services. Samples of our patients were however inconsistently tested and reported. Whenever possible additional samples were collected for testing in an international laboratory. From this experience it appears that systematic national testing in patients with diseases such as BU and yaws needs to be improved.

In summary, skin wounds represent a neglected health issue in rural West Africa. We provide here an estimate of the burden and of the clinical epidemiology. Most wounds were due to minor injuries, such as mechanical traumas and burns, and furuncles. These wounds have the potential to heal quickly if treated appropriately with basic wound management but are otherwise prone to complications, such as secondary bacterial infections, chronic wound development, sepsis, systemic diseases, and disability. Severe wounds requiring specific and more intensive treatment were mostly due to major injuries and skin NTDs, particularly BU. Children were the most affected group, and all major aetiologies were found more frequently in school-age children. However, comparison with previous studies shows that school-based surveys underestimate wound prevalence, because of selection bias due to schooling rates and social isolation caused by stigmatizing diseases. Our combined approach with health services-based and household-based surveys allowed us to detect many wounds that would have otherwise reached the attention of the health care system too late, if at all. Household-based surveys are resource-intensive and not sustainable on the long term or on a large scale, but community-based approaches with active detection strategies relying on community health workers can fill this gap and allow for early detection and treatment [12]. This is particularly important for skin NTDs, which, in contrast to declining incidence rates reported by national control programs, may suffer from substantial underreporting.

In the ongoing wound project, we will assess in repeated combined household- and health services-based surveys the impact of the community-based wound management model “Identify and treat wounds early” on the wound burden and clinical epidemiology.

## Supporting information

S1 Checklist STROBE Statement—checklist of items that should be included in reports of observational studies. (DOCX)
